# miR-99 inhibits cervical carcinoma cell proliferation by targeting TRIB2

**DOI:** 10.3892/ol.2013.1473

**Published:** 2013-07-17

**Authors:** JIA-XUAN XIN, ZHEN YUE, SHUAI ZHANG, ZHONG-HUA JIANG, PING-YU WANG, YOU-JIE LI, MIN PANG, SHU-YANG XIE

**Affiliations:** 1 Key Laboratory of Tumour Molecular Biology in Binzhou Medical University, Department of Biochemistry and Molecular Biology, Binzhou Medical University, Yantai, Shandong 264003, P.R. China; 2 Yantai City Hospital for Infectious Disease, Yantai, Shandong 264000, P.R. China; 3 Yantai Traditional Chinese Medical-Science (TCM) Hospital, Yantai, Shandong 264000, P.R. China; 4 Department of Epidemiology, Binzhou Medical University, Yantai, Shandong 264003, P.R. China

**Keywords:** microRNAs, cervical carcinoma, gene expression, cell proliferation, miR-99

## Abstract

MicroRNAs (miRNAs) have significant roles in cell processes, including proliferation, apoptosis and stress responses. To investigate the involvement of miR-99 in the inhibition of HeLa cell proliferation, an miR-99 gene expression vector (pU6.1/miR-99), which overexpressed miR-99 in HeLa cells after transient transfection, was constructed. The expression of miR-99 was detected by qPCR. Cell proliferation and apoptosis were analyzed by cell viability, proliferation and apoptosis assays, as well as by electron microscopy. The results showed that overexpression of miR-99 in HeLa cells increased the HeLa cell mortality rate. Moreover, miR-99 overexpression was able to markedly inhibit HeLa cell proliferation according to the 3-(4, 5-dimethylthiazol-2-yl)-2,5-diphenyltetrazolium bromide assay. The cell apoptosis rate was significantly higher in pU6.1/miR-99-treated cells compared with that in the control cultures. Increases in intracellular electron density, as well as the proportion of nuclear plasma, blebbing phenomena and apoptotic bodies were observed in pU6.1/miR-99-treated cells compared with control cultures according to electron microscopy analysis. The Tribbles 2 (TRIB2) 3′-untranslated region was also observed to be targeted by miR-99 and the results further demonstrated that miR-99 was able to negatively regulate TRIB2 expression in HeLa cells The results indicate that miR-99 acts as a tumor suppressor gene in HeLa cells, establishing a theoretical basis for its application in cancer therapeutics.

## Introduction

Cervical carcinoma is the second most frequently diagnosed cancer among females and is also one of the leading causes of cancer-associated mortality in females in developing countries (1). The mechanisms of cervical carcinoma formation remain largely unknown (2). At present, the first-line therapy is radical surgery with adjuvant chemotherapy. In recent years, along with increases in rate of diagnosis, improvements to surgical methods and the application of comprehensive therapy have caused improvements in the cure rates of cervical carcinoma. However, cervical carcinoma remains a significant health problem in both developed and developing countries. Therefore, it is important to identify a novel non-surgical targeting intervention or drug target for treating cervical carcinoma (3,4).

MicroRNAs (miRNAs) are a class of non-coding, single-stranded RNA molecules that are ~19–25 nucleotides in length and have key roles in the regulation of gene expression in plants and animals. They function as post-transcriptional gene regulators by pairing with the 3′-untranslated regions (3′-UTRs) of specific target messenger RNAs (mRNAs), resulting in their degradation or the repression of translation (5–8). miRNAs have been shown to be involved in cell growth, proliferation, apoptosis and stress responses (9,10). In addition, previous studies have indicated that numerous miRNAs are vital in cancer initiation and progression, through the regulation of their target gene pathways involved in cancer pathogenesis, as oncogenes or tumor suppressors (11–14). However, the role of miRNAs in regulating tumor metastasis has only been recently studied and there remains much to be investigated. It is important to study the association between target genes and miRNAs to understand the regulatory mechanism of miRNAs in animal development, tumor invasion and cell proliferation (6).

Tribbles (TRIBs) are a gene family which control the specificity of the activation of mitogen-activated protein kinases. A study demonstrated that rats treated with the TRIB2 gene contracted acute myeloid leukemia (AML) and the TRIB2 gene appears to be underexpressed when the growth of leukemia cells is inhibited (15). However, increases in the expression of TRIB2 were observed in AML patients (16,17). It has been demonstrated that TRIB2 induces AML through a series of mechanisms, including inhibiting C/EBPα (18). However, the biological role of TRIB2 in cervical carcinoma remains unclear.

The phenomenon of downregulation, as well as upregulation, of miRNAs is most frequently observed in cancer, suggesting that miRNAs function as tumor suppressor genes or oncogenes (19). It has been demonstrated that the miR-99 family regulates stress responses, apoptosis, proliferation and angiogenesis (20). Generally, miR-99 functions as a tumor suppressor gene. In the present study, we hypothesized that miR-99 binds to the 3′-UTR of TRIB2 to negatively regulate TRIB2 expression, and we constructed an miR-99 gene expression vector (pU6.1/miR-99) to investigate the effects of miR-99 on HeLa cell proliferation and apoptosis. The results demonstrated that miR-99, as a tumor suppressor gene, was able to induce HeLa cell apoptosis.

## Materials and methods

### Construction of miRNA expression vector (pU6.1/miR-99)

The miR-99 gene (NW_001794337) was amplified by PCR from human genomic DNA of human whole blood. Written informed consent was obtained from the patients. The forward primer was 5′-CATC GGATCCTACTATTGA AACAAAAGCAG-3′, while the reverse primer was 5′-ATCGAAGCTTCTATTGT TGAACGGCACT-3′. The amplification conditions were as follows: 5 min initial denaturation at 95°C followed by 28 cycles of 45-sec denaturation at 95°C, 30-sec annealing at 56°C and 45-sec elongation at 72°C. The obtained 363-bp fragment of miR-99 was cloned into the T-vector (Takara Bio, Inc., Otsu, Japan) to construct the T-miR-99 vector. Subsequently, the fragments were subcloned into pRNAi-U6.1/Neo (Biomics Biotechnologies, Nantong, China) using *Bam*HI and *Hin*dIII (Takara Bio, Inc.) restriction sites. The identity of the human miR-99 sequence in the plasmid was confirmed by using the constructed plasmid as a template for the generation of PCR and via automated DNA sequencing (Biosune, Shanghai, China; data not shown).

### Cell culture and miRNA-expressing plasmid transfection

HeLa cells (Shanghai Institute of Cell Biology, Shanghai, China) were maintained in RPMI 1640 medium (Gibco, Carlsbad, CA, USA) supplemented with 10% fetal calf serum (Hyclone, Logan, UT, USA) and 10 U/ml penicillin-streptomycin (Sigma, St. Louis, MO, USA). pRNAi-U6.1/Neo (Biomics Biotechnologies), using U6 promoter-driven green fluorescent protein (GFP) expression, was used as an miRNA expression vector. For cotransfection, cells were treated with 0.5 μg miRNA and 0.5 μg pcDNA-GFP-UTR using Lipofectamine 2000 (Invitrogen Life Technologies, Carlsbad, CA, USA) according to the manufacturer’s instructions. The GFP expression in cells was observed under a fluorescent microscope (BX43, Olympus, Inc., Japan) at 48 h after transfection. The percentage of positive cells was detected by flow cytometry (FACS, Beckman Coulter, Inc., Miami, FL, USA).

### Western blotting

The cells were collected at 72 h after transfection. Cell lysates were prepared in RIPA buffer (Beyotime Institute of Biotechnology, Haimen, China). The concentrations of the extracted protein were determined by BCA protein assay (Beyotime Institute of Biotechnology). Following SDS-PAGE, the samples were transferred onto nitrocellulose membranes (Millipore, Bedford, MA, USA). Following incubation with TBS containing 5% non-fat dry milk and 0.1% Tween-20 (TBST) with agitation at room temperature for 2 h, the membranes were incubated with the primary antibody (anti-TRIB2; Santa Cruz Biotechnology, Inc., Santa Cruz, CA, USA) at 4°C overnight, then washed three times with TBST. The membranes were incubated with the corresponding secondary antibody (1:3000; Beijing Zhongshan Golden Bridge Biotechnology Co., Ltd., Beijing, China) for 1 h at room temperature. Bands were visualized using an ECL kit (Millipore).

### RNA isolation and qPCR

Total RNA was isolated with TRIzol reagent (Invitrogen Life Technologies), then cDNA was synthesized and detected via reverse transcription-PCR (RT-PCR) and qPCR. The PCR products were analyzed by electrophoresis on a 1% agarose gel containing ethidium bromide. miRNA was isolated with the mirVana miRNA kit (Ambion, Austin, TX, USA). Following isolation, miRNAs were polyadenylated using poly(A) polymerase (Ambion). The cDNA was synthesized with an RT primer, 5′-AACATGTACAGTCCATGGATGd(T)30N(A, G, C or T)-3′. The forward primer of miR-99 used to amplify the miRNA was 5′-CCCGTAGATCCGATCTTGTG-3′, while the reverse primer was 5′-AACATGTACAGTCCATGGATG-3′. qPCR was then performed using SuperTaq Polymerase (Takara Bio, Inc.) according to the manufacturer’s instructions. The expression of miRNAs was detected with an RG3000 system (Corbett Research, Cambridge, UK) using the Quantitect SYBR-Green Kit (Qiagen, Hilden, Germany), as follows: initial denaturation at 95°C for 2 min, followed by 40 cycles of 95°C for 20 sec, 55°C annealing for 15 sec and extension at 72°C for 30 sec. Fluorescence was observed at 585 nm at each extension step at 72°C. Human 5S ribosomal RNA (rRNA) served as a control. The forward primer of 5S rRNA was 5′-GCCATACCACCCTGAACG-3′ and the reverse primer was 5′-AACATGTACAGTCCATGGATG-3′.

### Trypan blue exclusion test of cell viability

Tryplan blue is a dye that binds to DNA when the cell membrane is disrupted (dead cells), and it cannot enter into living cells with intact membranes (21). Briefly, HeLa cells (4×10^4^/well) were seeded in 24-well tissue culture plates (Corning Inc., Corning, NY, USA). Following transfection and stimulation, the cells were trypsinized and the cell pellets were collected by centrifugation at 1,806 × g for 2 min. Finally, the cells were resuspended in 50 μl medium per well and stained with 10 μl 0.4% Trypan blue solution for 4 min. Blue cells were counted as dead cells and the relative death rate was calculated as the number of dead cells divided by the total number of cells.

### Cell proliferation assay with 3-(4, 5-dimethylthiazol-2-yl)-2,5diphenyltetrazolium bromide

Cell proliferation was assayed with colorimetric MTT. Briefly, HeLa cells were seeded in 96-well plates. Cells were treated as indicated for 24 h, then 10 μl MTT was added to 100 μl culture media and cultured for 4 h at 37°C. Subsequently, 100 μl dimethyl sulfoxide was added to each well to dissolve the formazan completely. The optical density was detected at 570 nm on a microplate reader (Bio-Rad, Hercules, CA, USA).

### Flow cytometry analysis for cell apoptosis

At 48 h after transfection, the HeLa cells were harvested from the plates by trypsinization and collected by centrifugation at 1,806 × g for 2 min, then washed twice with PBS. The cells were then resuspended in 1 ml PBS and incubated with 1 ml propidium iodide at 4°C for 30 min, at a cell density of 1×10^6^. The cells were then analyzed using a flow cytometer (FACS, Beckman Coulter, Inc.).

### Electron microscopy analysis of HeLa cells

Following transfection for 48 h, the HeLa cells were trypsinized and centrifuged at 4515 × g for 2 min. The cell pellets were harvested and fixed with a 37°C solution of 2% paraformaldehyde, 2.5% glutaraldehyde (Ted Pella, Inc., Redding, CA, USA) in 0.1 M sodium cacodylate (pH 7.4) and incubated for an additional 30 min on ice. The cells were then rinsed three times for 3 min each with 0.1 M sodium cacodylate containing 3 mM calcium chloride (pH 7.4) on ice, and post-fixed with 1% osmium tetroxide, 0.8% potassium ferrocyanide and 3 mM calcium chloride (pH 7.4) for 1 h. The cultures were stained overnight with 2% uranyl acetate at 4°C and embedded in Durcupan resin (Fluka, St. Louis, MO, USA). Ultrathin (70-nm) sections were evaluated by transmission electron microscopy (EM; JEM-100cx; JEOL Ltd, Tokyo, Japan) operated at 80 kV. Images were recorded at a magnification of ×8,000.

### Statistics

SAS software (Chicago, IL, USA) was used to analyze the significance of all results and Student’s t-test was used for inter-group comparisons. P<0.05 was considered to indicate a statistically significant difference.

## Results

### Overexpression of miR-99 after treating HeLa cells with pU6.1/miR-99

**Figure 1:**
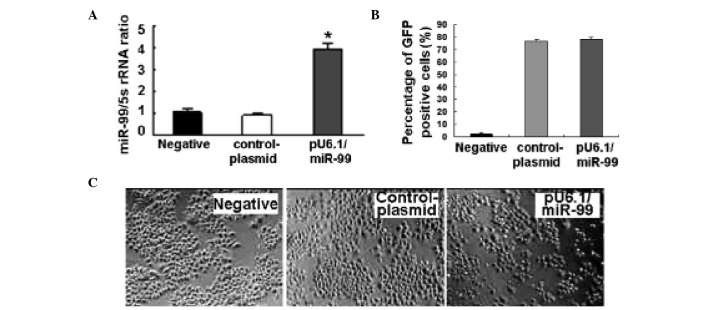
Effects of miR-99 on HeLa cell growth. (A) miR-99/5s rRNA ratio. qPCR showed that miR-99 was expressed effectively in HeLa cells following pU6.1/miR-99 transfection. (B) GFP-positive cells were used to reflect the plasmid transfection rate, which is shown to be >75%. (C) The number of living HeLa cells was lower in pU6.1/miR-99-treated cells compared with that in control plasmid-treated (pRNAi-U6.1/Neo) cultures. rRNA, ribosomal RNA; GFP, green fluorescent protein.

Initially, the quality of the isolated total RNA was tested by electrophoresis. The bands of 28S, 18S and 5S were clearly shown and not degraded. miR-99 was observed to be upregulated after HeLa cells were treated with the pU6.1/miR-99 vector, according to qPCR (Fig. 1A).

### Morphological changes following pU6.1/miR-99 treatment

After HeLa cells were transfected with the pU6.1/miR-99 vector that expresses GFP to reflect the transfection rate, the results showed that the transfection rate was >70% (Fig. 1B) and miR-99 was overexpressed in pU6.1/miR-99 cultures compared with the controls (Fig. 1A). The pU6.1/miR-99-transfected HeLa cells exhibited clear cell volume reduction, shrinkage and increasing numbers of floating cells, while the control cultures (pRNAi-U6.1/Neo-NC-treated cells) were polygonal or spindle shaped and adhered tightly to the well (Fig. 1C).

### Inhibiting HeLa cell proliferation by miR-99

**Figure 2:**
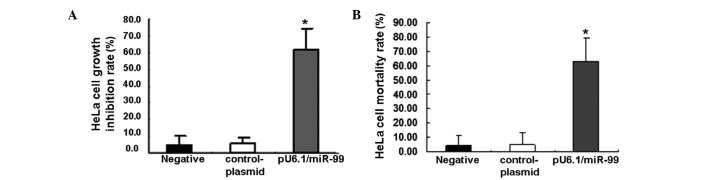
Cell proliferation and mortality were analyzed using MTT and Trypan blue assays. (A) HeLa cell growth was suppressed significantly by miR-99 following pU6.1/miR-99 transfection compared with that of the controls (P<0.01). (B) The Trypan blue results showed that there was a higher mortality rate in pU6.1/miR-99-treated cells compared with that of the controls (P<0.01). MTT, 3-(4, 5-dimethylthiazol-2-yl)-2,5-diphenyltetrazolium bromide.

The previously mentioned results demonstrated that the overexpression of miR-99 may suppress HeLa cell growth. To further investigate, HeLa cell proliferation was detected following treatment with the pU6.1/miR-99 plasmid using MTT. The results showed that the overexpression of miR-99 markedly inhibited cell proliferation in pU6.1/miR-99-treated cells compared with that in the control cells (Fig. 2A, P<0.01). A greater number of dead cells were also observed in pU6.1/miR-99-treated cultures when Trypan blue was used to reflect the cell mortality rate (Fig. 2B, P<0.01).

### Induction of HeLa cell apoptosis with miR-99

**Figure 3:**
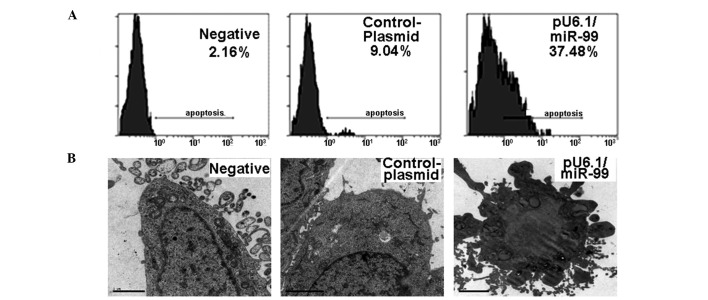
Apoptotic cells were detected by FACS and electron microscopy. (A) More apoptotic HeLa cells (~37.48%) were observed among pU6.1/miR-99 transfected cultures compared with that among the controls (2.16 or 9.04%). (B) Variations in cell morphology were observed by electron microscopy. Compared with the controls, pU6.1/miR-99-transfected cells showed increases in intracellular electron density and the proportion of nuclear plasma. The cell nucleus presented material densification or plaques.

To further study the effect of miR-99 on HeLa cell growth, cell apoptosis was analyzed by flow cytometry and electron microscopy following pU6.1/miR-99 treatment. The HeLa cell apoptotic rate was 37.48% in pU6.1/miR-99-treated cultures, which was significantly higher than that in pRNAi-U6.1/Neo-NC control (9.04%) or negative control cultures (2.16%) (Fig. 3A). Under electron microscopy, the negative control and pRNAi-U6.1/Neo-NC-treated cells showed rich microvilli on the cell surface, intact cells and nuclear membranes, visible bilayers, rich organelles in the cells and visible large numbers of rough endoplasmic reticulum and ribosomes in the cytosol. However, the pU6.1/miR-99-transfected cells showed increases in intracellular electron density and the proportion of nuclear plasma, as well as patchy nuclear material densification or plaques, while the nucleoli had almost disappeared (Fig. 3B). Blebbing phenomena and apoptotic bodies were also observed in pU6.1/miR-99-transfected cells (Fig. 3B).

### miR-99 suppresses TRIB2 expression in apoptotic HeLa cells

**Figure 4:**
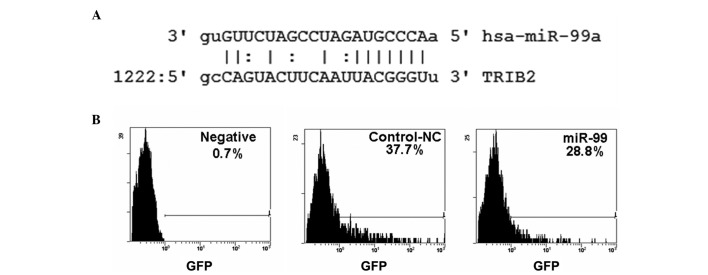
TRIB2 3′-UTR was targeted and regulated by miR-99. (A) The TRIB2 3′-UTR site was targeted by miR-99. (B) GFP expression was lower in miR-99 treated cells (28.8%) compared with that in the control-NC (miR-99-mutation)-treated cultures (37.7%), indicating that TRIB2 3′-UTR was regulated by miR-99. TRIB, Tribbles; 3′-UTR, 3′-untranslated region, GFP, green fluorescent protein.

**Figure 5:**
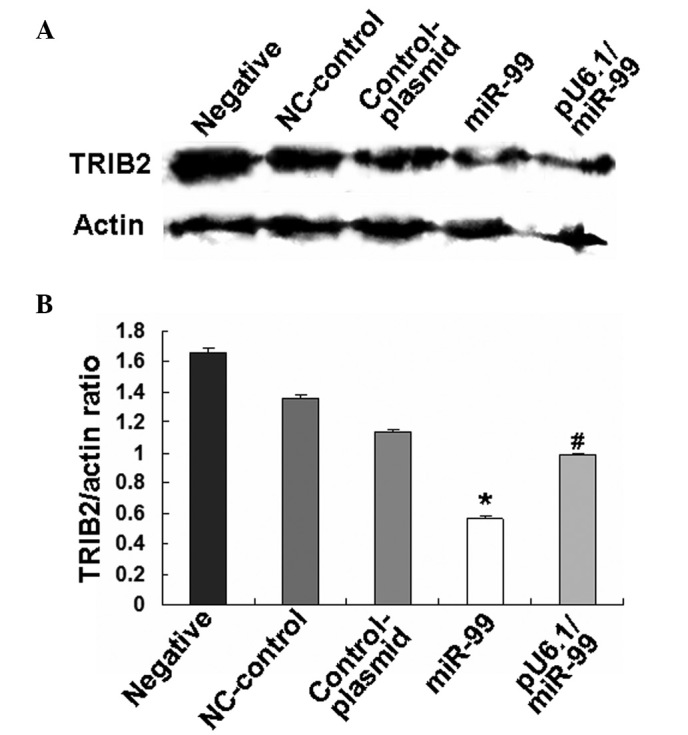
TRIB2 expression was detected by western blotting. (A) TRIB2 expression in the miR-99- and pU6.1/miR-99-treated cells was significantly lower than that of the NC (miR-99-mutation)- or pRNAi-U6.1/Neo-treated controls (^*^P<0.01, ^#^P<0.05). (B) Relative values for TRIB2 vs. actin are indicated. TRIB, Tribbles.

To investigate the mechanism by which apoptotic HeLa cells are induced by miR-99, the miR-99 targeting gene was predicted using microRNA Targetscan software (http://www.targetscan.org/index.html) and microRNA.org (http://www.microrna.org/microrna/getGeneForm.do) online. It was observed that TRIB2 was one of the genes targeted by miR-99 (Fig. 4A). Subsequently, the pcDNA-GFP-TRIB2-3′-UTR vector [which includes the TRIB2-3′-UTR and is described in our previous study (22)] was cotransfected with miR-99 into HeLa cells. The results showed that the number of GFP-positive cells was noticeably decreased in miR-99-treated cells compared with control plasmid-treated cultures (Fig. 4B), indicating that the TRIB2-3′-UTR was regulated by miR-99. Western blotting further demonstrated that the overexpression of miR-99 was able to down-regulate TRIB2 expression in HeLa cells following miR-99 or pU6.1/miR-99 vector treatment (Fig. 5).

## Discussion

miRNAs regulate signaling molecules through translational repression or gene splicing and modulate at least one-third of all human gene expression (23). miRNAs have important roles in cell proliferation and differentiation, and altered miRNA expression may lead to cancer. The majority of miRNAs are highly conserved and timing-, tissue- and cell-specific, as well as being subject to developmental, spatial and temporal regulation. miRNAs are also involved in cell differentiation, proliferation and apoptosis. Numerous studies have demonstrated that miRNAs are closely associated with the presence of a variety of tumors. Their targets may be tumor suppressor genes or oncogenes involved in tumorigenesis. In our previous study, >200 abnormal miRNA expression patterns were identified in DMC-induced apoptotic A549 cells. Further cluster analysis (by Cluster 3.0, CapitalBio Corporation, Beijing, China) and qPCR showed increases in the expression of miR-16, miR-34a, miR-34b, miR-34c, miR-17-5p and miR-125, whereas the expression levels of miR-106, miR-150, let-7c and miR-99 were decreased.

In the present study, the pU6.1/miR-99 expression vector, which could express miR-99 effectively in HeLa cells, was constructed first. Moreover, the overexpression of miR-99 induced HeLa cell apoptosis and inhibited HeLa cell proliferation following transfection with the pU6.1/miR-99 expression vector. The pRNAi-U6.1/Neo plasmid was selected to construct the pU6.1/miR-99 expression vector, due to two main advantages: i) it contains the neomycin gene, which make it is easy to establish stable cell lines by G418; and ii) it expresses GFP, which makes it is easy to observe its transfection rate. Additionally, these proteins do not affect cell growth and function.

The miR-99 family contains three members, miR-99a, miR-99b and miR-100 (24), which regulate cell stress responses, apoptosis, proliferation and angiogenesis. The expression of the miR-99 family has been observed to be increased following exposure to a single dose of DNA damage to induce the DNA repair pathways (25). It has been shown that miR-99a is able to change the efficiency of DNA repair by regulating SNF2H (26). Porkka *et al* reported that the expression of the miR-99 family was reduced in the majority of advanced prostate cancer compared with normal prostate epithelium (27). In the present study, it was demonstrated that miR-99 acted as a tumor suppressor gene and was able to induce HeLa cell apoptosis by regulating TRIB2 expression.

The TRIB genes were first identified in Drosophila (28) and comprise a family of kinase-like proteins containing a single kinase-like domain. Mammals have three homologs of TRIB, TRIB1, TRIB2 and TRIB3 (29,30). It has been observed that TRIB2 is highly expressed in several acute myeloid leukemias lacking C/EBP-α mutations (16). TRIB2 causes fatal transplantable AML when introduced in murine hematopoietic stem cells *in vivo*(31). In a previous study, we showed that TRIB2 was an oncogene, which was more highly expressed in lung adenocarcinoma compared with paracancerous tissue controls (22). In the present study, using microRNA Targetscan software, the TRIB2 3′-UTR was observed to be targeted by miR-99 and the results further demonstrated that miR-99 was able to negatively regulate TRIB2 expression in HeLa cells.

In conclusion, the present study studied miR-99 expression and its roles in regulating HeLa cell proliferation at the cellular level, and demonstrated that the overexpression of miR-99 induced HeLa cell apoptosis. The study identified that miR-99 has a tumor suppressor function, suggesting a theoretical basis for its application in cancer therapeutics.

## Abbreviations

miRNAs: microRNAs
mRNA: messenger RNAs
AML: acute myeloid leukemia
RT-PCR: reverse transcription-PCR
MTT: 3-(4,5-dimethylthiazol-2-yl)-2,5-diphenyltetrazolium bromide
DMSO: dimethyl sulfoxide
TRIB: Tribbles
